# Deep-amplicon sequencing of the complete beta-tubulin gene in *Trichuris trichiura* before and after albendazole treatment

**DOI:** 10.1016/j.ijpddr.2024.100570

**Published:** 2024-11-12

**Authors:** Javier Gandasegui, Berta Grau-Pujol, Valdemiro Novela, Osvaldo Muchisse, Maria Cambra-Pellejà, Anélsio Cossa, José Carlos Jamine, Charfudin Sacoor, Eric A.T. Brienen, Francesc Catala-Moll, Lisette van Lieshout, María Martínez-Valladares, Roger Paredes, José Muñoz, Stephen R. Doyle

**Affiliations:** a Wellcome Sanger Institute, Cambridgeshire, United Kingdom; b Barcelona Institute for Global Health (ISGlobal), Hospital Clínic - University of Barcelona, Barcelona, Spain; c Manhiça Health Research Centre (CISM), Maputo, Mozambique; d Mundo Sano Foundation, Buenos Aires, Argentina; e GraphenicaLab S.L., Barcelona, Spain; f Facultat de Medicina I Ciències de La Salut, Universitat de Barcelona (UB), Barcelona, Spain; g Leiden University Center for Infectious Diseases. Parasitology Research Group, Leiden University Medical Center (LUMC), the Netherlands; h IrsiCaixa, Badalona, Spain; i Instituto de Ganadería de Montaña (CSIC-Universidad de León), León, Spain; j Infectious Disease Networking Biomedical Research Center, Centro de Investigación Biomédica en Red de Enfermedades Infecciosas (CIBERINFEC), Carlos III Health Institute, Madrid, Spain; k Universitat de Vic-Universitat Central de Catalunya, Vic, Spain; l Department of Infectious Diseases, Hospital Universitari Germans Trias I Pujol, Badalona, Spain; m Center for Global Health and Diseases, Department of Pathology, Case Western Reserve University School of Medicine, Cleveland, OH, USA

**Keywords:** *Trichuris trichiura*, Deep-amplicon sequencing, Beta-tubulin, Benzimidazole resistance, Genomics

## Abstract

Concerns about the emergence of benzimidazole resistance in soil-transmitted helminths (STH) infections, particularly against *Trichuris trichiura*, have arisen. Previous studies of veterinary nematodes have linked benzimidazole resistance to single-nucleotide polymorphisms (SNPs) at three specific codons in the beta-tubulin gene, but similar associations in STH have not been consistently observed. In this work, we screened the complete beta-tubulin gene previously linked to benzimidazole resistance in *T. trichiura* by deep-amplicon sequencing to identify genetic variants and associate levels of diversity with drug response to albendazole. We used 99 DNA samples extracted from *T. trichiura* pooled eggs, previously semi-purified from human stool samples collected in Manhiça district, Mozambique. We obtained a set of 39 amplicons of the complete gene by subjecting the pooled eggs to long-read PCR and subsequently sequencing them. Of those amplicons, 22 and 17 were obtained from stool samples collected before, and 21 days after albendazole treatment, respectively. We observed genetic variation across the whole gene sequence, in both exons and introns; however, none were associated with the previously proposed resistance-associated SNPs, and none were predicted to significantly affect protein function. No significant differences in genetic diversity were observed between pre- and post-treatment samples. Using publicly available genome-wide data, we also analysed a second beta-tubulin isotype in the *T. trichiura* genome. We focused on detecting the canonical SNPs and assessing for signatures of genetic selection around this second isotype gene. This analysis did not reveal evidence supporting this second isotype's role in anthelmintic resistance. Despite the limitations of our study, such as a small sample size, particularly paired pre- and post-treatment samples (n = 6), or a restricted geographical area, we found no evidence linking either of the two beta-tubulin genes to benzimidazole resistance in *T. trichiura*, suggesting that genetic markers of drug resistance likely exist outside the beta-tubulin genes.

## Highlights

• First deep-amplicon sequencing of the complete beta-tubulin of *Trichuris trichiura*.
• No genetic variants were observed at codons 167, 198 and 200 of beta-tubulin.
• Other variants identified are not predicted to affect protein function.
• There was no difference in the genetic diversity before and after treatment.
• A second isotype assessed using genomics data was not associated with resistance.

## Graphical abstract

**Figure:**
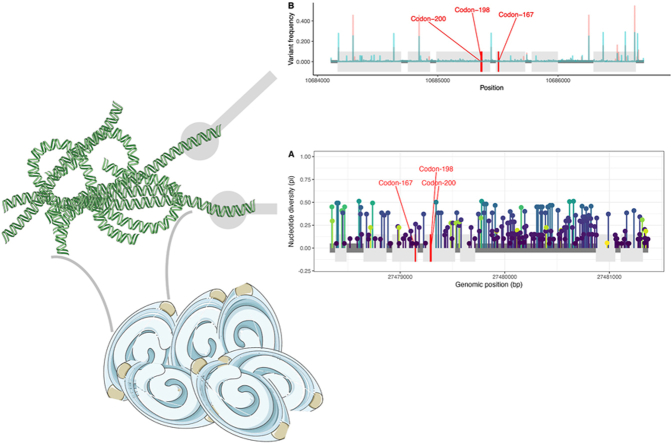

## 1 Introduction

Soil-transmitted helminths (STH), including the roundworm *Ascaris lumbricoides*, the whipworm *Trichuris trichiura*, and two hookworms, *Necator americanus* and *Ancylostoma duodenale*, are major human pathogens that infect over 1.5 billion people worldwide and are responsible for tremendous disease burdens and disability (World Health Organization, 2020a, 2020b). The current World Health Organization (WHO) mandated strategy for STH control has focused on morbidity control through mass drug administration (MDA) of the benzimidazole-class anthelmintics albendazole (ALB) and mebendazole, mainly to pre-school and school-aged children and women of reproductive age (Montresor et al., 2020). However, there are increasing concerns that the ongoing success of these programs is at risk due to the almost complete reliance on a single drug treatment strategy. The use of ALB to control *T. trichiura* is suboptimal relative to the treatment efficacy of other helminths (Moser et al., 2017), and in recent years, the efficacy of benzimidazoles against STH has decreased, particularly against *T. trichiura* (Moser et al., 2017; Schulz et al., 2018). One hypothesis is that the widespread and frequent use of benzimidazole monotherapy has led to the selection of anthelmintic-resistant parasites. If true, the evolution of resistance to MDA treatment by *T. trichiura* is a significant threat to progress to control STH and plans to interrupt transmission (Freeman et al., 2019).

Benzimidazole resistance in veterinary gastrointestinal nematodes, primarily those infecting ruminants, has been associated with single-nucleotide polymorphisms (SNPs) in the beta-tubulin gene that causes an amino acid substitution at codons 167, 198 or 200 (Kwa et al., 1995; Silvestre and Cabaret, 2002; Ghisi et al., 2007; Martínez-Valladares et al., 2020; Doyle et al., 2022a). The strong association between these variants and resistance prompted the investigation of homologous beta-tubulin genes of STH to identify if similar variants were also associated with treatment responses. After an initial report that observed an increase in the frequency of the putative resistant variant at codon 200 in *T. trichiura* after ALB treatment (Diawara et al., 2013), subsequent studies have not observed such a relationship between the presence or frequency of any putative benzimidazole resistance SNP and drug efficacy (Matamoros et al., 2019; Grau-Pujol et al., 2022; Levecke et al., 2024). In some cases, a clear absence of a genetic response was reported, describing careful, robust sampling before and after treatment of the same individual (Grau-Pujol et al., 2022). The growing evidence of a lack of genetic association between these canonical SNPs and treatment response suggests that other genetic variants different from these three SNPs in the beta-tubulin gene could be involved in drug resistance.

In this work, we have expanded a previous evaluation of the role of the putative benzimidazole resistance SNPs in the three codons (167, 198 and 200) in ALB efficacy in the Manhiça district, Southern Mozambique (Grau-Pujol et al., 2022) by screening the complete beta-tubulin gene to identify putative novel genetic variants associated with drug response. To do so, we subjected DNA from pooled eggs of *T. trichiura*, collected before and after ALB treatment, to deep-amplicon sequencing to identify variation within the complete beta-tubulin gene previously linked to benzimidazole resistance in *T. trichiura* (Diawara et al., 2013). Finally, we also assessed the potential role of genetic variation and drug resistance of the second isotype of the beta-tubulin gene in the *T. trichiura* genome using genome-wide data previously published (Doyle et al., 2022b).

## 2 Material and methods

### 2.1 Ethics approval and consent to participate

The study was performed according to the Declaration of Helsinki (version of Fortaleza, Brazil, October 2013), current ICH-GCP guidelines and all applicable national and local regulatory requirements (Spanish Royal Decree 1090/2015). National Bioethics Committee for Health in Mozambique approved this study (Ref.:517/CNBS/17). Participation in this study was voluntary. We obtained written informed consent in either Portuguese or Changana from all study participants older than 18 years. Caregivers provided written informed consent for participants under 18 years. Participants between 15 and 17 years old also provided written informed assent. For illiterate caregivers, informed consent was obtained in the presence of a literate witness independent of the study.

### 2.2 Study population, sample collection and processing

This work builds upon a previous study that assessed the efficacy of ALB on STH in the Manhiça district. A detailed description of the study design, study area, participant recruitment, sample collection, and processing has been described elsewhere (Grau-Pujol et al., 2021, 2022). Briefly, sample collection was conducted as follows: after consent to participate, two stool samples were collected per participant on two consecutive days. These samples were transported in a cold box to the Manhiça Health Research Centre (CISM) for laboratory analysis. Participants with at least one STH infection detected by microscopy at CISM were asked to provide a third pre-treatment stool sample on the day they received ALB 400 mg. A stool sample was also collected 21 days post-treatment to assess treatment response. Both pre- and post-treatment samples were screened for STH presence by the Kato-Katz method and qPCR, and subsequently, positive samples underwent egg concentration using metallic sieves, as previously described (Grau-Pujol et al., 2022). All sediments retained after egg concentration were stored at −80 °C until DNA extraction. DNA extraction was performed using the QIAamp PowerFecal Pro DNA Kit (QIAGEN, Hilden, Germany) following the manufacturer's instructions at the University of León, Spain. The DNA samples were stored at −20 °C until use.

To analyse the complete beta-tubulin gene of *T. trichiura* by deep-amplicon sequencing, we used 99 DNA samples that tested positive for *T. trichiura* by microscopy or qPCR that were available in our laboratory, 55 of which were collected before treatment and 44 after treatment.

### 2.3 PCR amplification and sequencing

We designed primers targeting the complete beta-tubulin gene of *T. trichiura*. Using the DNA sequence from the NCBI database (GenBank Accession No. AF034219.1), we located the beta-tubulin gene in the *T. trichiura* genome via the BLASTN tool in WormBase ParaSite (WBPS). We extracted this DNA sequence from WBPS, including an additional 1000 bp flanking on either side of the beta-tubulin gene, which was used for primer design in Primer3 (Untergasser et al., 2007). The forward primer sequence used for PCR amplification was CGGACCGCAGCTCATTTCAT, and the reverse primer sequence was TACACCCAACAGTCCCCAAC, targeting an amplicon size of 2611 bp. DNA was amplified using the Platinum SuperFi II PCR Master Mix (Thermo Fisher Scientific) according to the manufacturer's instructions. Long-range PCR conditions were 98 °C for 30 s, followed by 35 cycles of 98 °C for 10 s, 60 °C for 30 s and 72 °C for 60 s, followed by 5 min at 72 °C and 4 °C to finish. PCR products were monitored via 1% agarose gel electrophoresis; if the initial amplification was unsuccessful, PCR products were subjected to a second PCR using the same conditions. Amplified products were purified using a SpeedTools Clean-up kit (BioTools).

Specific PCR amplicons were sequenced as follows: amplified DNA templates were purified from non-DNA molecules, and Illumina sequencing adapters and dual indices were attached using the Nextera XT index Kit (Illumina Inc.), followed by a corresponding PCR amplification program as described in the MiSeq 16S rRNA Amplicon Sequencing protocol. After the second round of purification, amplicon libraries were quantified using a Quant-iT™ PicoGreen® dsDNA Assay Kit (Invitrogen) and diluted in equimolar concentrations (4 nM) for further pooling. Sequencing was performed on an Illumina MiSeq™ platform (Illumina Inc.) using the paired-end 300 base-length protocol at the genomics core facility in the Germans Trias i Pujol research campus in Badalona, Spain.

### 2.4 Sequencing data processing and analysis

#### 2.4.1 Mapping

Sequencing reads were mapped using a *Nextflow* mapping pipeline called *mapping-helminth* v.1.0.8. Briefly, paired-end FASTQ sequencing files were first converted to merged unaligned BAM files using *GATK* v.4.1 (Van der Auwera and O’Connor, 2020). Then, Illumina adapters were marked, and the unaligned BAM files were converted to interleaved single FASTQ files to be mapped to the *T. trichiura* reference genome (assembly ID TTRE3.0, available from https://parasite.wormbase.org/Trichuris_trichiura_prjeb535/Info/Index/) using *minimap2* v.2.16. (Li, 2016). Then, unaligned and aligned reads were merged and sorted using *GATK*, and *sambamba* v.0.6.6 was used to mark duplicate reads (Tarasov et al., 2015). Finally, mapped reads were combined using *samtools merge* v.1.14. (Danecek et al., 2021). *Multiqc* v.1.17. was used to generate a report on the mapping process (Ewels et al., 2016).

#### 2.4.2 Analysis of variation in the beta-tubulin gene

We identified genetic variants from the BAM files obtained after mapping using *Grenedalf* v.2.1., a tool for analysing pooled sequencing data (Czech et al., 2024). Variants identified by *Grenedalf* were retained using a manually curated BED file with the beta-tubulin gene coordinates in the *T. trichiura* reference genome. First, we obtained the per-site variations and the number of reads for each nucleotide (reference, alternative, and total counts). From this information, we compared the coverage and variant frequency for all samples. Then, we split the data into pre- and post-treatment groups to identify variants that were more frequent in post-treatment samples and potentially related to treatment response and drug resistance.

We also evaluated the potential impact of these variations using *SnpEff* (Cingolani, 2022). First, we obtained a VCF file after SNP calling and genotyping using standard procedures. Variant calling was performed using *GATK* v.4.1. Initially, variants were identified, and GVCF files were generated for each BAM file using the *GATK HaplotypeCaller* tool. Then, variants were consolidated, and GVCF files were merged using *GATK CombineGVCFs* before joint-call cohort genotyping with *GATK GenotypeGVCFs*. Finally, a single multisample VCF file containing all variants and samples was generated. Then, we selected the variants in the VCF file based on *Grenedalf* results; we selected variants with a minimum frequency of 10% in at least one sample to be considered reliable for further analysis with *SnpEff*. For this purpose, we used the annotation available in WBPS to predict the effect of variants on the protein-coding sequence. The gene predictions of this annotation were made by the Parasite Genomics group at the Wellcome Sanger Institute, as previously described (Foth et al., 2014). Additionally, we used *Grendalf* to estimate the per-sample nucleotide diversity (pi) in the beta-tubulin gene and compared it to STH treatment phenotype data, including eggs per gram (epg) of stool from the Kato-Katz method and the Ct-values obtained from qPCR. We assessed the correlation between these variables using Spearman's rank correlation test. Finally, we compared the nucleotide diversity between samples collected before and after treatment using the Kruskal-Wallis test.

#### 2.4.3 Evaluating the second beta-tubulin isotype in drug resistance

Using previously published genome-wide data, we explored the potential role of a second beta-tubulin isotype in the *T. trichiura* genome (Doyle et al., 2022b). We included *T. trichiura* data from specimens collected from baboons (n = 2) and humans, and among them, we included ancient *T. trichiura* samples collected in Europe (n = 17), as well as modern specimens collected in China (n = 10), Honduras (n = 8), and Uganda (n = 12). A thorough description of the specimens, variant calling, and filtering can be found in the original work (Doyle et al., 2022b). The nucleotide variation and diversity analysis in this beta-tubulin gene was performed using vcftools and a bed file of exon coordinates derived from manual gene curation.

#### Availability of data and materials

Participant data cannot be shared publicly because of participants’ consent, but data will be available on a reasonable request from the Data Management external unit at ISGlobal (bioesdm@isglobal.es). Raw sequencing data used in this study are publicly available from the Sequence Read Archive under the BioProject ID: PRJNA1145892. The code used to generate and analyse data and figures can be found at https://github.com/Gandasegui/deep_amp_trich.

## 3 Results and discussion

In veterinary medicine, the canonical SNPs at codons 167, 198 and 200 in the isotype-1 of the beta-tubulin gene in gastrointestinal nematodes, such as *Haemonchus contortus* and *Teladorsagia circumcincta*, have been widely linked to benzimidazole resistance (Kwa et al., 1995; Silvestre and Cabaret, 2002; Ghisi et al., 2007). Consequently, recent research has focused on the investigation of the potential role of these genetic variants in beta-tubulin genes of STH species in the development of anthelmintic resistance, even if some STH species, such as *T. trichiura*, are genetically divergent from intestinal parasitic worms of ruminants (Doyle, 2022). Moreover, a recent study comparing the beta-tubulin isotypes of all STH species with other parasites found that the *T. trichiura* beta-tubulin under study (misidentified as isotype-1 in previous works) diverges significantly from other beta-tubulins previously associated with benzimidazole resistance (Roose et al., 2021). Considering these differences, previous pyrosequencing (Grau-Pujol et al., 2022; Levecke et al., 2024) and Sanger sequencing (Matamoros et al., 2019) experiments did not replicate the genetic association between treatment response and the canonical SNPs in *T. trichiura*. Thus, we have evaluated whether other variants present in the beta-tubulin gene can cause loss of drug efficacy using a deep-amplicon sequencing method, which offers significant advantages over conventional sequencing methods such as Sanger sequencing and pyrosequencing. It provides sequencing data with precise quantification of variant frequencies and the ability to capture sequence diversity in heterogeneous samples, which enhances the overall resolution and sensitivity of genomic analyses. This approach has been pivotal in studying the epidemiology and drug resistance of other infectious diseases (Lerch et al., 2017; Santa et al., 2021; Francis et al., 2024; Lapp et al., 2024).

From the 99 pooled egg samples initially submitted to long-range PCR, we obtained DNA amplicons of the expected size of ∼2600 bp from 46 samples, which were subsequently purified and sequenced. Afterwards, sequencing reads in paired FASTQ files obtained from each sample were mapped against the *T. trichiura* reference genome. Out of the 46 samples, seven showed a very low percentage of mapped reads (Table S1), which entailed that those seven purified amplicons did not correspond to the beta-tubulin gene, resulting in a final set of 39 sequencing datasets obtained from 33 participants. Out of the 39 amplicons, 16 were obtained from participants before the administration of ALB, 11 amplicons were obtained from participants sampled 21 days after treatment, and 12 amplicons (six pre- and six post-treatment) were paired in terms that each pair was collected from the same participant. A brief description of the population characteristics and the Kato-Katz and qPCR results can be found in Table 1.

**Table 1:** Description of study participants’ characteristics and the infection intensity expressed by mean EPG and Ct-values.

	Overall	Pre-treatment	Post-treatment	Paired^a^
**Number of participants (n (%))**	33	16	11	6
**Mean age (SD)**	24.45 (24.53)	21.44 (22.28)	24.64 (26.04)	32.17 (30.08)
**Male (n (%))**	15 (45.5)	9 (56.2)	5 (45.5)	1 (16.7)
**Administrative unit (n (%))**				
**3 De Fevereiro**	5 (15.2)	4 (25.0)	1 (9.1)	0 (0.0)
**Calanga**	10 (30.3)	3 (18.8)	5 (45.5)	2 (33.3)
**Ilha Josina Machel**	3 (9.1)	1 (6.2)	2 (18.2)	0 (0.0)
**Maluana**	10 (30.3)	6 (37.5)	1 (9.1)	3 (50.0)
**Manhica Sede**	5 (15.2)	2 (12.5)	2 (18.2)	1 (16.7)
**Positive by Kato-Katz (n (%))**	28 (71.8^b^)	12 (75%)	7 (63.6)	pre - 5 (83) post - 4 (66.6)
**Mean EPG (range)**	278.8 (6–5310)	532 (6–5310)	133.7 (12–540)	pre - 76.8 (18–234) post - 25.5 (12–60)
**Positive by qPCR (n (%))**	33 (100)	16 (100)	11 (100)	pre - 6 (100) post - 6 (100)
**Median Ct-value (range)**	27.2 (24–33.7)	27.9 (24–30.8)	28.2 (25.3–33.7)	pre - 27 (25.4–28.7) post - 28 (26–32)

a This column corresponds to the 12 paired amplicons obtained from 6 participants. For the intensity of infection expressed as EPG and Ct-value, the results for the pre- and post-treatment samples are shown.

b This percentage has been estimated in the 39 amplicons (16 pre-treatment, 11 post-treatment and 12 paired samples).

**Fig. 1:**
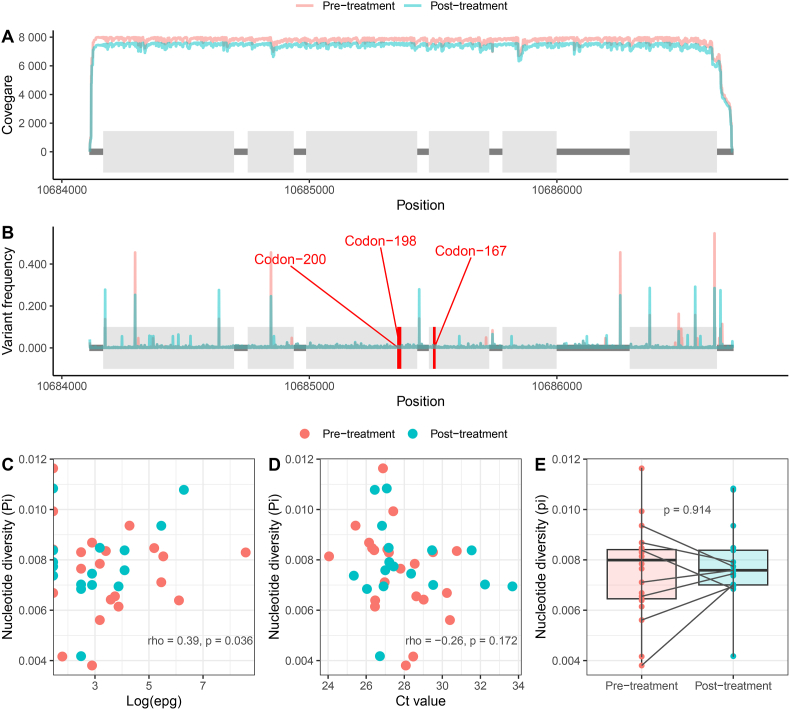
Analysis of the genetic variation and nucleotide diversity of the beta-tubulin gene of Trichuris trichiura before and after albendazole treatment. A) and B), Gene structure of the beta-tubulin gene, with exons presented as grey boxes separated by black lines representing introns and the position in the Trichuris_trichiura_1_001 is expressed in the x-axis. A) Mean coverage and B) mean variant frequency for pre and post-treatment samples in the y-axis; the red lines represent the positions of canonical resistance-associated variants at codons 167, 198, and 200, at which no variation was identified here. Correlation between C) the eggs per gram of stool (left panel) and D) Ct-values (right panel) and nucleotide diversity; the Spearman correlation coefficient and p-value are shown on the plot. D) Boxplot of the nucleotide diversity and comparison between pre- and post-treatment samples (a line links paired staples); the Kruskal-Wallis p-value is shown on the plot. (For interpretation of the references to colour in this figure legend, the reader is referred to the Web version of this article.)

We first estimated the coverage and the frequency of all the variants in the amplicon (Table S2). Then, we compared the difference between samples collected pre- and post-treatment to explore potential sequencing artefacts that can affect variant identification. We did not observe relevant differences in the coverage along the whole gene between pre and post-treatment samples (Fig. 1A). We observed variation along the entire beta-tubulin gene (Fig. 1B), particularly because Grenedalf reported variations of at least one read (Czech et al., 2024). To identify biologically relevant variants and remove potential PCR or sequencing errors, we selected those with at least 10% frequency in at least one sample, resulting in 39 variants, 29 located in the exons and 10 in the introns. None of the canonical resistance-associated SNPs were found. Then, we evaluated the potential impact of these variants on the transcript. According to *SnpEff*, all SNPs caused synonymous amino acid changes, and/or none are predicted to have a significant effect on protein function (Table S3), concluding that the beta-tubulin sequence was similar to those previously published and identical between the samples collected before and after treatment in this study. Although there were no variants related to drug resistance in the complete gene, we explored if the genetic information extracted from this gene can be used to observe evidence of genetic selection by the treatment. We estimated the nucleotide diversity (Pi) in the complete beta-tubulin gene and correlated it with the infection intensity and treatment response. We do observe a direct correlation between nucleotide diversity and egg count (rho = 0.39; p-value = 0.036) (Fig. 1C), whereas no correlation was observed between Pi and the Ct-values provided by the qPCR (rho = −0.26; p-value = 0.172) (Fig. 1D). These analyses suggest that samples with larger egg counts exhibit higher genetic diversity, as expected. However, this correlation was not observed when using Ct-values as a measure of infection intensity. This finding supports the notion that qPCR results should be interpreted only as a rough indication of the intensity of infection and Ct-values cannot be directly translated to the number of eggs in the stool (Papaiakovou et al., 2019; Cools et al., 2020). We also compared the nucleotide diversity in the complete gene between pre- and post-treatment samples, where we did not find relevant differences (p-value = 0.91), with particular attention to the paired samples that did not exhibit an overall tendency of a reduction in diversity (Fig. 1E). We acknowledge the reduced sample size, particularly the low number of paired samples collected from the same participant, as a relevant limitation of this study. However, neither the observed genetic variations nor the genetic diversity suggest even a weak relationship between the drug response and the beta-tubulin gene. Collectively, our results further support the alternative hypothesis that this gene cannot produce or is related to drug resistance, either by the canonical SNPs or other variants in the gene. Therefore, it would be plausible to consider that markers of benzimidazole resistance could be located in different regions of the genome.

**Fig. 2:**
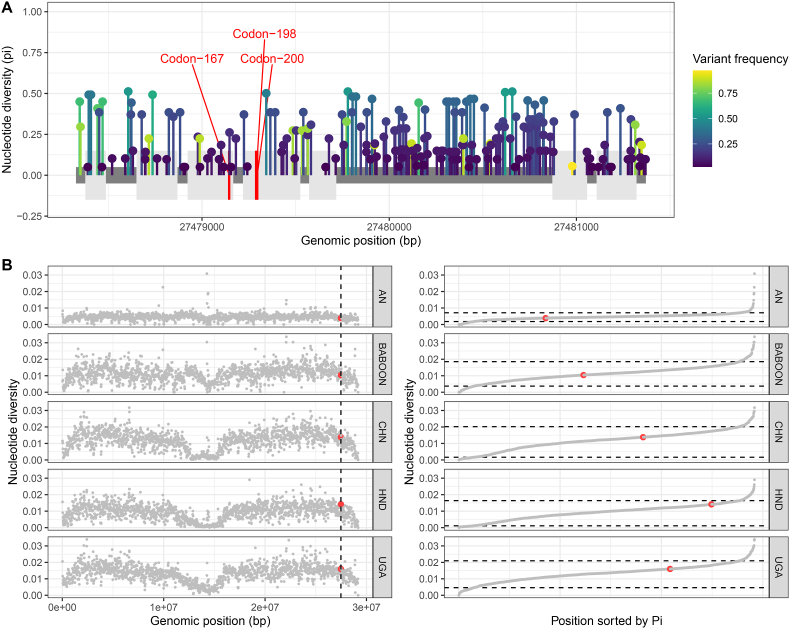
Analysis of variation within and surrounding the second isotype of the beta-tubulin gene. A) Gene structure of the second isotype of the beta-tubulin gene, with exons presented as grey boxes separated by black lines representing introns. Coloured lines indicate variants identified; the nucleotide diversity is shown in the y-axis, whereas the colour shows the variant allele frequency. The red lines represent the positions of canonical resistance-associated variants at codons 167, 198, and 200, at which no variation was identified. B) Nucleotide diversity (measured in 20 kb non-overlapping windows) in the Trichuris_trichiura_2_001 scaffold containing the second isotype of the beta-tubulin gene. The left panel indicates the gene's position in the chromosome by the black vertical line and red point in each population. The right panel indicates the ranked order of 20 kb windows based on nucleotide diversity values; the upper and lower dashed lines represent the 95th and 5th quantiles of the distribution, respectively. The gene (red point) is highlighted, demonstrating its level of nucleotide diversity, which is not deemed an outlier relative to the observed genetic diversity throughout the scaffold. (For interpretation of the references to colour in this figure legend, the reader is referred to the Web version of this article.)

To evaluate the potential role of the second beta-tubulin isotype in drug resistance, we used genome-wide data from a previously published global population structure analysis of human and non-human infective *T. trichiura*, including specimens found in archaeological dig sites and latrines associated with Viking settlements (Doyle et al., 2022b). This sample cohort includes parasites collected from areas with high drug pressure due to extensive historical records of MDA campaigns or from the study areas where the ALB efficacy was found to be extremely low (a recent clinical trial found an ALB efficacy of 4.2% in the same study area in Honduras) (Matamoros et al., 2021). We observed 204 polymorphic sites along this second isotype of the beta-tubulin gene, including 60 within the exons. However, none of these variants was found in canonical codons 167, 198 or 200 (Fig. 2A). We then evaluated the nucleotide diversity of the genomic region where the second beta-tubulin is placed in the context of the chromosomal genetic diversity; however, there was little evidence of broader-scale genetic change on standing genetic variation in the region surrounding the beta-tubulin gene that might be associated with positive selection on a gene within that region. We even observed this locus to be in areas with higher nucleotide diversity in populations with historical benzimidazole drug pressure due to MDAs (Fig. 2B). This second isotope of the beta-tubulin has been described as the most divergent beta-tubulin compared to the isotype-1 of the beta-tubulin gene of benzimidazole resistance parasites (Roose et al., 2021). Our results show no evidence to support specific variants or selection in this second beta-tubulin gene of *T. trichiura* and anthelmintic resistance. Additionally, the work by Doyle and colleagues conducted a similar analysis on the first isotype of the beta-tubulin gene, leading to similar results (Doyle et al., 2022b). This supports that there would be no evident link between these beta-tubulin genes and anthelmintic resistance in *T*. *trichiura*.

It is worth noting that a systematic nomenclature for beta-tubulin isotypes within Clade I of nematodes would be necessary to accurately reflect evolutionary relationships and avoid confusion across species. Historical variations in naming have led to assumptions about homology that may not be valid, as seen with the mislabelling of isotypes in *Ascaris* spp. compared to other nematodes like *H*. *contortus* and *Caenorhabditis elegans* (Roose et al., 2021). Establishing a uniform naming convention will not only improve clarity in research but also support more precise studies on drug resistance mechanisms. However, conducting the thorough phylogenetic analysis necessary for such a standardized nomenclature within Clade I nematodes lies beyond the scope of this manuscript. Future work should focus on creating a robust classification system that aligns with the growing knowledge of nematode phylogeny.

In summary, we did not observe any genetic variants or selection based on the beta-tubulin gene. The absence of genetic variants associated with resistance could be attributed to several factors. One possible explanation is that ALB treatment does not select for resistant parasites, and the differences in treatment response may be due to other factors, such as variations in ALB pharmacokinetics (Whittaker et al., 2022) or microbiome composition (Schneeberger et al., 2022; Appiah-Twum et al., 2023). However, considering the global variability in benzimidazole efficacy, not only in *T. trichiura* but also in other STH species (Matamoros et al., 2021; Hürlimann et al., 2022; Welsche et al., 2023), the hypothesis of genetically diverse parasite populations with different drug response phenotypes seems better to explain the local and global disparities in treatment outcomes. Another explanation for the lack of evidence of genetic selection is the presence of a single, drug-resistant parasite population in the study area, which aligns with the previously observed low ALB efficacy in the study area (previous reports showed a cure rate of ALB below 10% when estimated by qPCR in the same study area) (Grau-Pujol et al., 2022). Finally, the absence of evidence could be also due to the limitations of using the beta-tubulin gene as a target sequence for assessing genetic selection based on nucleotide diversity beyond this gene, as it may not be information-rich enough. Previous research has highlighted the limitations of using small genetic regions in studying genetic diversity when compared with genome-wide approaches, which provide higher resolution and power to detect fine-scale population structure and subtle genetic differences that may be missed by smaller regions (Gilleard and Redman, 2016).

Nonetheless, our study is consistent with the previously reported absence of the canonical SNPs in the beta-tubulin gene not only in *T. trichiura* but also in other whipworm species (Hansen et al., 2013;Gandasegui et al., 2021; Rivero et al., 2024). Furthermore, recent studies on ascarids, including *A. lumbricoides*, *Ascaris suum*, and *Parascaris* spp., have similarly shown no presence of canonical SNPs in the beta-tubulin isotypes potentially associated with benzimidazole resistance (Roose et al., 2021; Özben et al., 2022; Jones et al., 2024). Although earlier research in STH suggested that these mutations might indicate resistance (Diawara et al., 2013; Furtado et al., 2019, 2020; Mendes de Oliveira et al., 2022), this has not yet been demonstrated for *T. trichiura*, *A. lumbricoides*, or *N. americanus*. It has been a recurring assumption that the presence of these SNPs alone could imply drug resistance, even without supporting phenotypic data. Another common assumption has been that resistance mechanisms identified in Clade V nematodes (such as *H. contortus* and *C. elegans*) could directly apply to Clade II species like *Ascaris* spp. or Clade I species like *Trichuris* spp, implying a shared genotypic mechanism across phylogenetically distant nematodes. This emerging body of evidence emphasizes the need to identify alternative mechanisms underlying benzimidazole resistance in STH.

Comprehensive studies employing genome-wide data to investigate anthelmintic resistance in STH are currently being carried out (Bär et al., 2024; Levecke et al., 2024). Genome-wide approaches could be essential for studying anthelmintic resistance in STH, as the resistance mechanisms may, in fact, be more complex and multifaceted than previously appreciated (Doyle and Cotton, 2019). Using whole genome sequencing data in helminthology has highlighted significant genetic diversity and divergence among helminth populations (Sallé et al., 2019). This diversity means that resistance may arise from multiple genetic loci and interactions, which are not fully captured by targeted gene studies, such as studies focused only on the beta-tubulin gene. By employing genome-wide strategies with appropriate sampling and metadata, we may gain a deeper and more precise understanding of anthelmintic resistance, ultimately leading to more effective control measures and treatment protocols for STH infections.

## 4 Conclusion

Our study reveals no relationship between the two beta-tubulin genes and benzimidazole resistance in *T. trichiura*, suggesting that other factors may influence treatment response. Our findings indicate that genetic markers for drug resistance likely exist outside the beta-tubulin gene. This research underscores the need for comprehensive, robust methodologies to understand better anthelmintic resistance mechanisms in soil-transmitted helminths. This is crucial for improving global control programs and preventing anthelmintic resistance emergence.

## CRediT authorship contribution statement

**Javier Gandasegui:** Writing – review & editing, Writing – original draft, Visualization, Resources, Methodology, Investigation, Formal analysis, Data curation, Conceptualization. **Berta Grau-Pujol:** Writing – review & editing, Methodology, Conceptualization.**Valdemiro Novela:** Writing – review & editing, Methodology, Conceptualization. **Osvaldo Muchisse:** Writing – review & editing, Project administration, Methodology, Investigation. **Maria Cambra-Pellejà:** Methodology, Investigation. **Anélsio Cossa:** Writing – review & editing, Methodology, Investigation. **José Carlos Jamine:** Project administration, Methodology, Investigation. **Charfudin Sacoor:** Methodology, Methodology, Investigation. **Eric A.T. Brienen:** Methodology, Investigation. **Lisette van Lieshout:** Methodology. **María Martínez-Valladares:** Writing – review & editing, Methodology, Investigation. **José Muñoz:** Methodology, Investigation, Conceptualization, Methodology, Conceptualization. **Stephen R. Doyle:** Writing – review & editing, Writing – original draft, Visualization, Supervision, Methodology, Investigation, Formal analysis.

## Declaration of competing interest

The authors declare that they have no known competing financial interests or personal relationships that could have appeared to influence the work reported in this paper.
